# Verification of neuroprotective effects of alpha-lipoic acid on chronic neuropathic pain in a chronic constriction injury rat model

**DOI:** 10.1515/biol-2021-0026

**Published:** 2021-03-12

**Authors:** Junhao Wang, Zhaohui Lou, Haiyang Xi, Zhi Li, Lepeng Li, Zhenzhen Li, Kai Zhang, Tetsuya Asakawa

**Affiliations:** Department of Orthopedic Surgery, First Affiliated Hospital of Zhengzhou University, Zhengzhou, Henan 450052, China; Institute of Clinical Medicine, First Affiliated Hospital of Zhengzhou University, Zhengzhou, Henan, China; Department of Neurosurgery, Hamamatsu University School of Medicine, Handayama, 1-20-1, Higashi-ku, Hamamatsu City, Shizuoka 431-3192, Japan; Research Base of Traditional Chinese Medicine Syndrome, Fujian University of Traditional Chinese Medicine, Fuzhou 350122, China

**Keywords:** neuropathic pain, peripheral nerve injury, alpha-lipoic acid, satellite glial cells, dorsal root ganglia

## Abstract

Treatment of neuropathic pain is far from satisfactory. This study sought evidence of a neuroprotective effect of alpha-lipoic acid (ALA) to treat neuropathic pain in a chronic constriction injury (CCI) rat model. A total of 48 rats were randomly divided into sham, CCI, or CCI + ALA groups. Mechanical and thermal nociceptive thresholds were evaluated as behavioral assessments. Dorsal root ganglia cells were assessed morphologically with hematoxylin and eosin staining and for apoptosis with P53 immunohistochemical staining. Compared with the sham group, the CCI group had a shorter paw withdrawal threshold and paw withdrawal latency, abnormal morphologic manifestations, and increased numbers of satellite glial cells and P53+ cells. These changes were significantly reversed by treatment with ALA. Our study indicates neuroprotective effects of ALA on chronic neuropathic pain in a CCI rat model. ALA is potentially considered to be developed as a treatment for neuropathic pain caused by peripheral nerve injury, which requires further verification.

## 1 Introduction

Peripheral nerve injury (PNI) is commonly caused by crush injury or mechanical trauma, leading to pain characterized by hyperalgesia and allodynia [1]. Spontaneous pain is frequent and may be intolerable. Although this is not a life-threatening condition, it seriously diminishes quality of life and is a frequent reason for doctor visits. Neuropathic pain is common in the general population. Approximately 80% of adults at some time or other complain of lower back or leg pain, but current therapy cannot completely prevent pain or relieve the consequences of neurologic damage [2,3]. PNI is therefore an important and increasing public health concern.

Mechanisms underlying PNI are complicated and not fully understood. Currently, there is increasing focus on the pathogenesis of dorsal root ganglia (DRG) damage. A recent study reported that peripheral neuropathic pain is caused by abnormal spontaneous activity of both damaged and undamaged DRG [4]. The DRG are sensitive to mechanical stimuli, with the adventitial coat being a very sensitive receptor for mechanical trauma. Under certain pathologic conditions such as trauma or inflammation, DRG are stimulated and cause pain, as the ganglia have no effective blood-nerve barrier. The DRG neuroglia are composed of satellite glial cells (SGCs) [5]. Neurons in the sensory ganglia are surrounded by SGCs, which play crucial roles in the initiation and promotion of peripheral neuropathic pain [6,7]. Previous studies demonstrated that SGCs can be triggered and activated by cytokines during the inflammatory response [8,9], which is often closely associated with neuropathic pain. Activation of SGCs is therefore considered a hallmark of PNI.

Alpha-lipoic acid (ALA) exhibits a strong neuroprotective effect. It is used to treat diseases caused by oxidative stress (OS) such as diabetic neuropathy [10] and multiple sclerosis [11] and to alleviate the inflammatory response [12]. It is also used as a modulator of various inflammatory signaling pathways [13]. As early in 2008, Melli et al. reported that ALA is neuroprotective in sensory neurons and prevents apoptosis of DRG cells [14]. A later study found that ALA reduces expression of oxides and increases antioxidants in sciatic nerve crush injury rat models [15]. We thus wondered whether ALA would have neuroprotective effects in PNI. In the present study, we employed chronic constriction injury (CCI) in rats as a model of chronic neuropathic pain [16] and P53 as a cellular apoptosis marker. The aim was to look for evidence of neuroprotective effects of ALA in CCI rat models, potentially leading to a novel treatment for neuropathic pain associated with PNI.

## 2 Materials and methods

### 2.1 Animals

A total of 48 male Sprague-Dawley rats (240 ± 20 g, age 2 months) were used. All rats were housed and fed at room temperature (23°C) with a humidity of 55 ± 15% and a 12 h light/dark cycle (lights on at 7:00 a.m.). Food and water were provided *ad libitum*. The animals were randomly divided into three groups. The dose of ALA (50 mg/kg) was selected according to the previous rat studies [17,18]. In the sham group (*n* = 16), the right sciatic nerve was exposed without any other treatment. The CCI group (*n* = 16) underwent nerve injury as described below and had a sham injection (0.9% saline solution, 50 mg/kg) every day after surgery. The CCI + ALA group (*n* = 16) also underwent nerve injury, and the rats were then treated with ALA (50 mg/kg) injections every day after surgery immediately prior to conducting behavioral assessments.


**Ethical approval:** The research related to animal use has been complied with all the relevant national regulations and institutional policies for the care and use of animals and was approved and supervised by the Animal Care and Use Committee of the First Affiliated Hospital of Zhengzhou University.

### 2.2 Surgical procedures

The CCI models were established according to the methods described in a previous study [16]. Briefly, the rats were anesthetized with tiletamine/zolazepam (Zoletil WK001, Virbac, France), 50 mg/kg intraperitoneally. The right sciatic nerve was exposed at the level of the mid-thigh, and the connective tissue around the nerve was cleared. Four ligatures were then tied around the nerve at 1–2 mm intervals using 4-0 chromic gut suture material.

### 2.3 Behavioral assessment

To assess the pain, mechanical and thermal nociceptive thresholds were examined as previously described [19]. To examine the mechanical nociceptive threshold, animals were placed individually into a small plastic cage with an open wire mesh bottom. Before testing, rats were left in the test cages for 30 min until their grooming and exploratory behaviors ceased and all four paws were placed on the bottom. Von Frey filaments (North Coast Medical Inc, Morgan Hill, CA, ranged from 1.08 to 40 g) were applied vertically to the planter surface of the paw with an upward force just sufficient to bend the microfilament. One of the tactile-defensive behaviorals, namely, brisk paw withdrawal was observed. We recorded the stimulus intensity at the time of foot and leg reactions, including foot reflexes, leg-stripping, and leg-turning, at intervals of 10 s per stimulus. Filaments were used in ascending order. Each filament was used once prior to advancing to the next filament. The smallest filament that conducted a paw withdrawal response was considered the threshold stimulus. The paw withdrawal threshold (PWT) index was recorded and averaged over five measurements. For thermal nociceptive thresholds, animals were placed in an acrylic box with a transparent glass plate and irradiated with radiant heat on both hind paws. The leg lift avoidance time (the time it took to respond to the thermal stimulus) was counted as the paw withdrawal latency (PWL). A low power (40 mW/cm^2^) intensity, a 20-second cutoff time, and a heating rate of 1°C/s were chosen according to the previous study [19]. The stimulus interval was 5 min, and the average of three measurements was calculated.

The behavioral assessments were performed 1 day before surgery and 3, 7, 14, and 21 days postsurgery. Animals were kept in the testing chambers for 30 minutes before each measurement so as to ensure they were accustomed to the test environment. After the behavioral tests, all animals were submitted to the morphological experiments.

### 2.4 Morphology

DRG tissues were examined morphologically for evidence of nerve damage with hematoxylin and eosin (HE) staining and for cellular apoptosis with P53 immunohistochemical staining.

Rats in each group were anesthetized with tiletamine/zolazepam (50 mg/kg) either on day 7 (*n* = 8) or day 21 (*n* = 8) after surgery. They were transcardially perfused with saline, followed by 250 mL of 4% paraformaldehyde (PH0427, Phygene, Fujian, China) in phosphate-buffered saline (PBS, 0.1 M, pH 7.4). DRG tissues from L4 to L6, corresponding to sciatic afferent fibers, were removed and fixed with 4% paraformaldehyde in PBS (pH 7.4) for 4 h. After rinsing, the DRG tissues were processed and embedded in paraffin. Subsequently, 5 µm thick sections were cut using a microtome with disaposable stainless knives (Yamato Kohki Industrial, Saitama, Japan). Some tissue sections were examined after standard HE staining. The remaining sections were submitted to P53 staining. To determine P53 immunoreactivity, anti-P53 mouse monoclonal antibodies (1:100, ZM-0408, ZSGB-BIO, Beijing, China) were applied to the sections overnight at 4°C. Secondary antibodies conjugated to horseradish peroxidase (SPN-9001, anti-rabbit; SPN-9002, anti-mouse, ZSGB-BIO, Beijing, China) were then applied for 15 min at 37°C as per the manufacturer’s instructions. Diaminobenzidine color reagent (SN640500, Celnovte Biotechnology, Henan, China) was used to develop color.

The SGCs surrouding the neurons were identified using the methods described by a previous study [20]. Briefly, all the sections were observed and quantitatively analyzed using a slice-analyzing system including an optical microscope (Nikon Ni-E, Japan) and the Image J 1.80 software package. DRG neurons were divided into large (>50 µm), medium (30–50 µm), and small (10–30 µm) with visible nucleoli. The numbers of surrounding SGCs and P53-immunopositive (P53+) cells in each of eight consecutive sections were calculated using the Scion Image 4.0.3 software package as following steps: All areas of the DRG were snapped for the following image analysis using at least sixty images in each group. After acquisition of the images, they were transformed into TIFF format (1,080 × 800 pixels). The processes of the images included converting the image to black and white, subtracting the background, elevating contrast, reducting the noise, and finally perfoming measurements. In this study, we set all DRG areas (ipsilateral to injury) as the regions of interest (ROI). A standard Erode and Dilate filter was employed to reduce the background noise. Dilate was used in the cell area to obtain a value comparable to the area before applying the Erode filter. Image binarization was performed to correct filter operations. Areas of objects presenting in the ROI were included in a measurements unit [20], which were set as 20,000 pixels, corresponding to 9,965 µm^2^ according to a presvious study [21]. Using these methods, the edematous DRG neurons and surrounding SGCs were identified. All these processes were performed by the same experienced researcher blinded to the treatments of groups.

### 2.5 Statistic analysis

Data were analyzed using SPSS software (V 19.0, IBM, USA). All data are reported as mean ± standard deviation. All statistical results were tested on both sides. Normality of distribution and homogeneity of variance tests were performed first. Analysis of variance followed by Bonferroni *post hoc* correction was then selected for multiple comparisons. *P* < 0.05 was considered to be statistically significant.

## 3 Results

**Figure 1:**
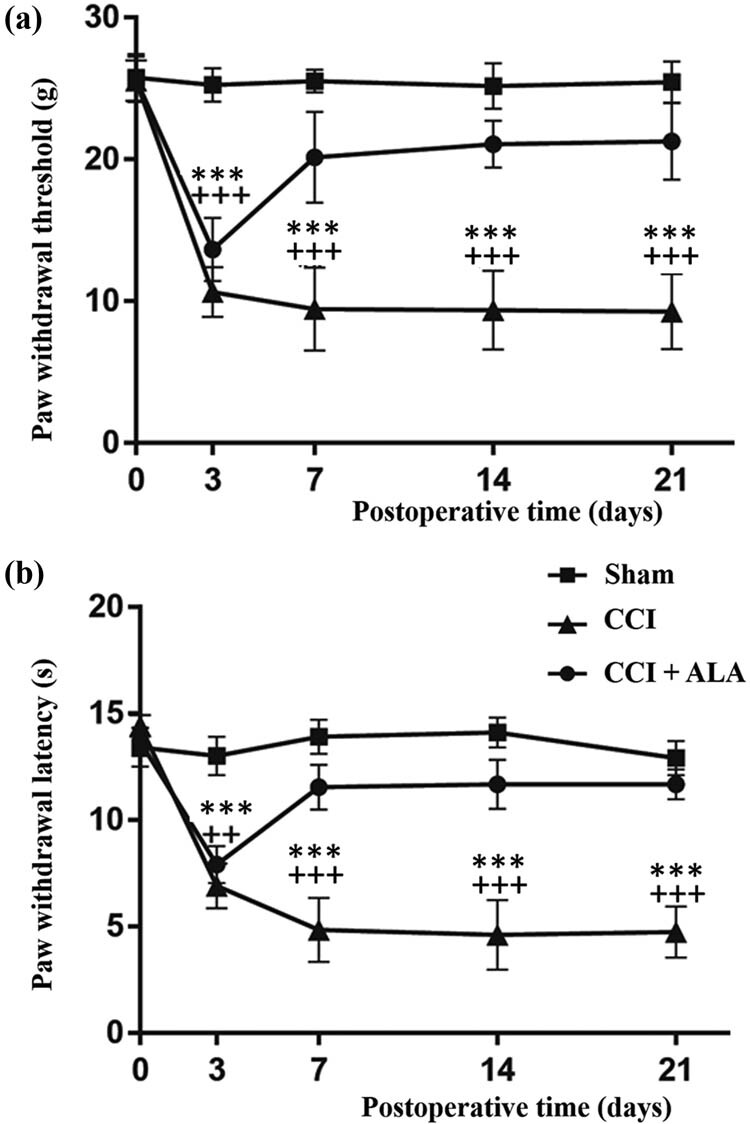
Changes in paw withdrawal thresholds (PWTs) and paw withdrawal latency (PWL) induced by chronic constriction injury (CCI) and its treatment with alpha-lipoic acid (ALA). (a) The PWTs in the CCI group were significantly lower than those in the sham group, whereas ALA treatment significantly enhanced PWTs, although not to the level of the sham group. (b) Similar to the PWTs, the PWLs of the CCI group were significantly lower than those of the sham group. Treatments with ALA significantly enhanced the PWLs, although not to the level of the sham group. ** means *P* < 0.01, *** means *P* < 0.001, sham vs CCI; ++ means *P* < 0.01, +++ means *P* < 0.001, CCI vs CCI + ALA.

CCI treatment significantly shortened the PWT (Figure 1a) and PWL (Figure 1b) as compared with the sham group. These changes were partly ameliorated by ALA treatment, although not to the normal levels of the sham group. The same differences between groups were present at postoperative days 3, 7, 14, and 21 (Figure 1).

**Figure 2:**
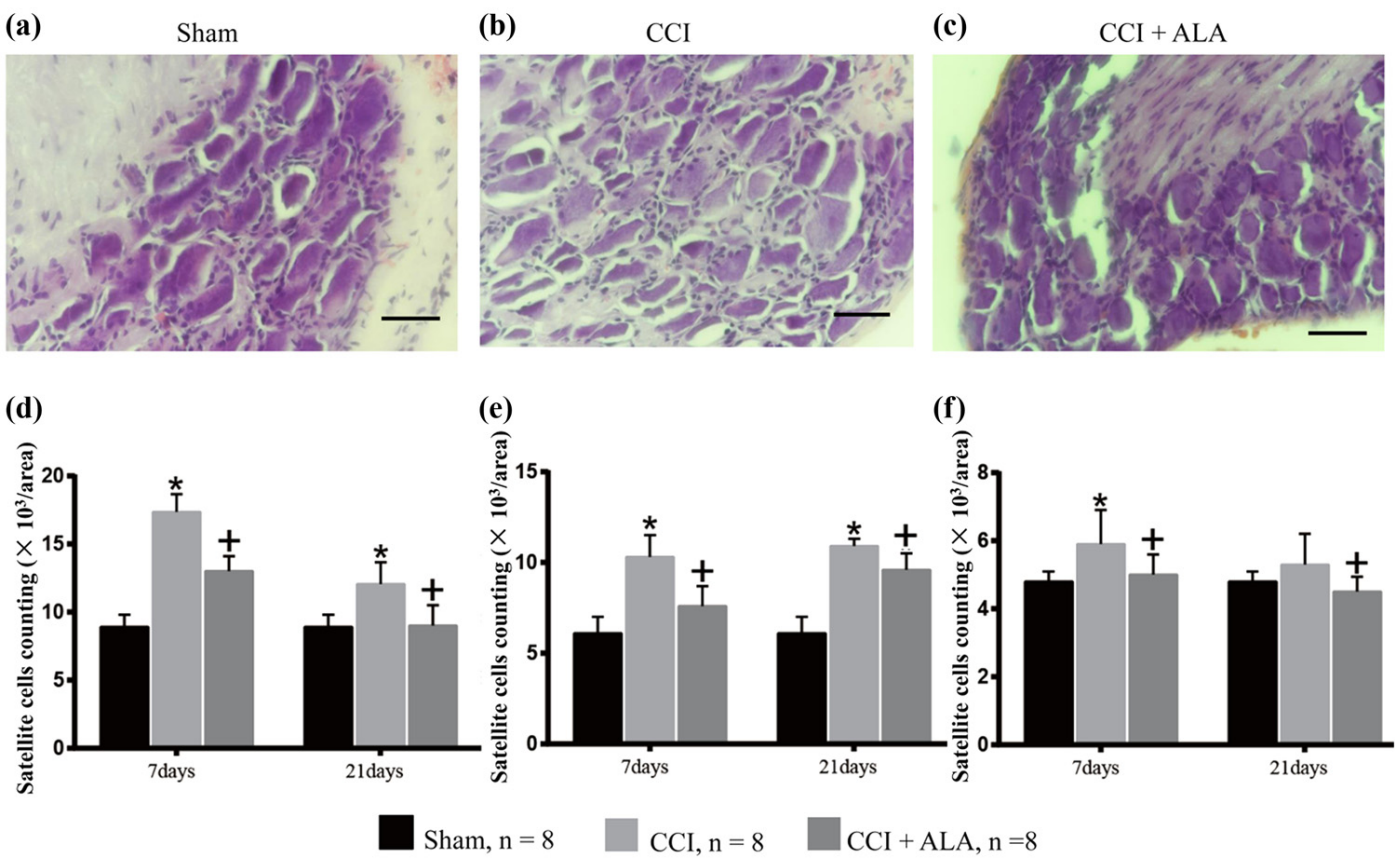
Morphology of dorsal root ganglia (DRG) and satellite glial cells (SGC) (hematoxylin and eosin). (a) Representative photo of DRG cells in the sham group with normally shaped neuronal cells. (b) Representative photo of DRG cells in the CCI group. Edematous neurons and the enhanced SCGs surrounding the neurons can be observed. (c) Representative photo of DRG cells in the CCI + ALA. Although there are some edematous neurons and enhanced SGCs, they are fewer than in the CCI group. (d–f) SGC cells counts/area surrounding large (>50 µm, d), medium (30–50 µm, e), and small (10–30 µm, f) DRG neurons. The results were analogous; cell numbers/area were significantly higher in the CCI group (vs sham group) and could be reduced after ALA treatment (vs CCI + ALA group). * means *P* < 0.05, sham vs CCI; + means *P* < 0.05, CCI vs CCI + ALA; scale bar = 50 μm; magnification: 40×; one area = 20,000 pixels = 9,965 µm^2^.

In the sham group, DRG cells appeared morphologically normal (Figure 2a). No edema or abnormal aggregation or proliferation of cells was observed. In the CCI group, the cell bodies of DRG neurons were markedly edematous with evident vacuolar-like changes (Figure 2b). The cytoplasm was concentrated, and the cell body volume contracted and deformed. Occasionally, apoptotic neuronal cells were observed, and the number of SGCs was obviously enhanced. Such changes indicated an enhanced inflammatory reaction associated with CCI.

In the CCI + ALA group, there was less neuronal cell body edema and fewer vacuolar changes than in the CCI group, but ALA treatment did not completely restore the tissue to a normal appearance (Figure 2c).

Counting the numbers of SGCs/area yielded similar results. Here, we set one area as 20,000 pixels, corresponding to 9,965 µm^2^. The numbers of SGS/area in the CCI group were significantly higher than those in the sham group (Figure 2d, surrounding large DRG neurons over 50 µm, at both 7 and 21 days after operation; Figure 2e, surrounding medium DRG neurons [30–50 µm], at both 7 and 21 days after operation; Figure 2f, surrounding small DRG neurons [10–30 µm], at 7 days after operation). ALA treatment partially relieved this effect, although without restoring the DRG tissues to normal at both 7 and 21 days after operation. This suggests that ALA treatment partly relieves the inflammatory reaction induced by CCI (Figure 2d–f).

**Figure 3:**
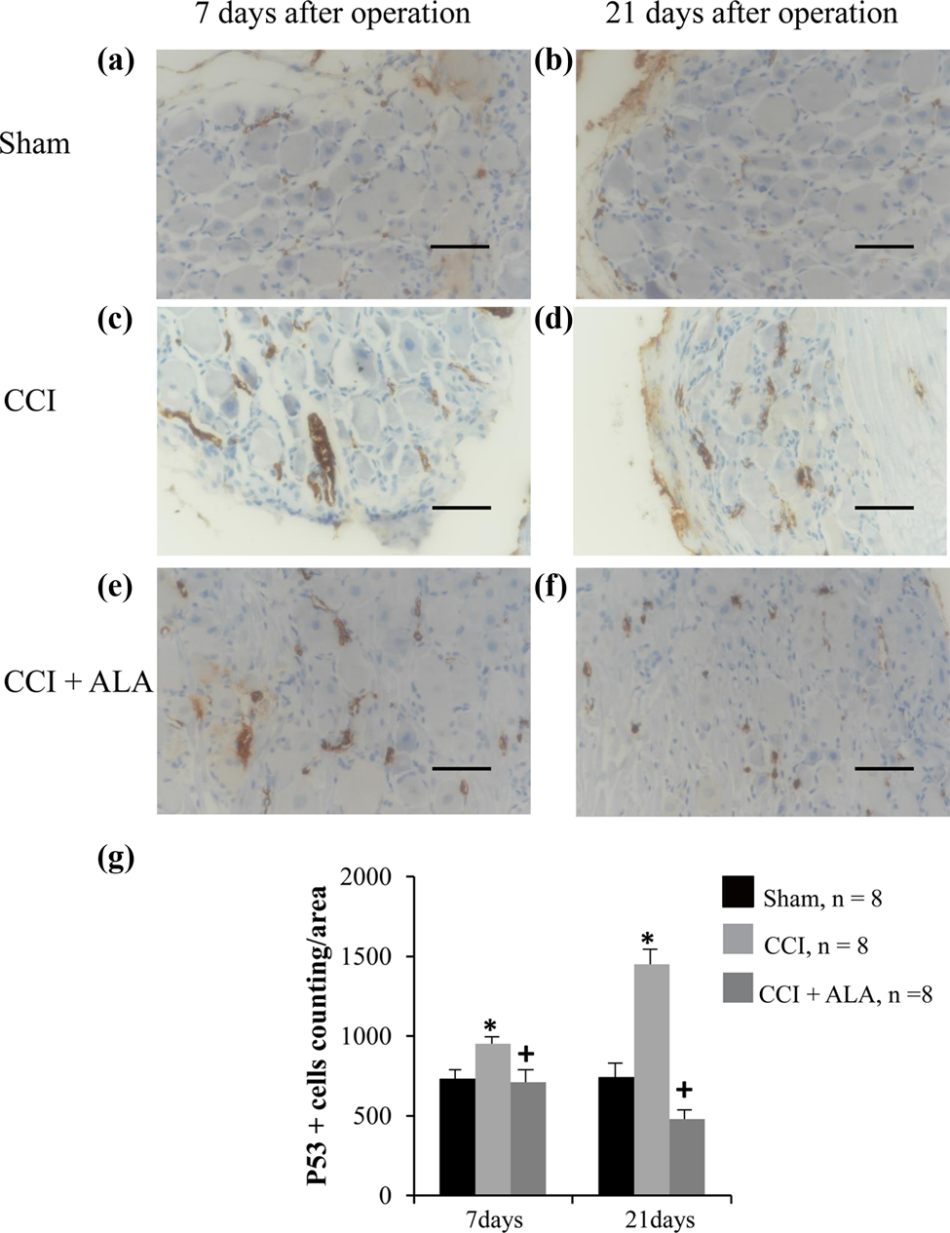
P53 immunohistochemistry of dorsal root ganglia (DRG) after chronic compression injury (CCI). Representative photo of P53 staining of DRGs in the sham group, (a) 7 and (b) 21 days after operation. The brown dots represented P53+ cells, which are rare in the sham group. Representative photos of P53 staining in CCI group, (c) 7 and (d) 21 days after operation. P53+ cells are significantly increased, even more so at 21 days than at 7 days after operation. The brown areas indicate apoptosis cells, which are small and shrunken. Representative photos of P53 staining in CCI + ALA group, (e) 7 days after operation and (f) 21 days after operation. There are fewer P53+ cells at both 7 and 21 days after operation than in the CCI group. (g) P53+ cell counts/area. CCI treatment significantly increased the number of P53+ cells, whereas ALA treatment led to normal numbers. * means *P* < 0.05, sham vs CCI; + means *P* < 0.05, CCI vs CCI + ALA; scale bar = 50 μm; magnification: 40×; one area = 20,000 pixels = 9,965 µm^2^.

Likewise, one area was set as 20,000 pixels, corresponding to 9,965 µm^2^. Compared with the sham group (Figure 3a and b), the numbers of P53+ cells/area were significantly higher in the CCI group (Figure 3c and d), suggesting that apoptosis was induced by CCI. The findings were similar at both 7 days (Figure 3c) and 21 days (Figure 3d) postoperatively. Treatment with ALA significantly reduced the number of P53+ cells/area compared with the CCI group (Figure 3), indicating that ALA can relieve CCI-induced apoptosis at both 7 days (Figure 3e) and 21 days (Figure 3f) after the operation.

## 4 Discussion

The present study used ALA to treat chronic neuropathic pain in a CCI rat model. We found that ALA treatment significantly shortened PWT and PWL, improved morphologic changes in the DRG neurons, reduced the aggregation and proliferation of SGCs, and decreased numbers of P53+ cells. Our findings have therefore provided behavioral (Figure 1), morphologic (Figure 2), and immunohistochemical (Figure 3) evidence that we successfully established a CCI-induced PNI model in rats. We also demonstrated that ALA treatment partially relieved the CCI-induced pathologic changes. We have therefore verified the protective effects of ALA on chronic neuropathic pain. These findings imply that ALA can be considered as a potential therapy for PNI-associated neuropathic pain, although further verification is needed.

The CCI rat model used in the present study is a classic model used to mimic chronic neuropathic pain [22]. Sciatic nerve ligation damages the peripheral nerve, so that the animal may try to bite the injured leg off. This abnormal behavior is regarded as a reaction to neuropathic pain [22,23]. In measurements of mechanical and thermal nociceptive thresholds, the PWT as well as PWL may shorten because of changes such as hyperpathia caused by CCI. Our behavioral data pointed a significant decreases in PWT and PWL after CCI, in accordance with a previous study [24]. Along with the morphologic and immunohistochemical evidence, our findings confirmed that we successfully established this CCI rat model. ALA treatment prolonged the PWT and PWL in the treatment group, indicating that it reduced hyperpathia, thus confirming the efficacy of ALA treatment.

Differing from astrocytes or oligodendrocytes, SGCs are a distinct type of glial cells that surround sensory neurons in ganglia of the peripheral nervous system. The role of SGCs is not fully understood, although studies have indicated they play a crucial role in neuropathic pain, including visceral [25] and peripheral neuropathic pain [26,27]. A number of studies have demonstrated that a variety of injurious stimuli may trigger activation of SGCs [27,28,29]. SGC activation is regarded as a neurophysiologic reaction to neuronal stress induced by these stimuli [26]. Activated SGCs commonly have remarkable cellular proliferation [28]. HE staining in our CCI rat models showed proliferated and abnormally aggregated SGCs. Along with other abnormal morphologic manifestations in the DRG neurons, we confirmed that the CCI procedure induces neurologic damage. ALA treatment reduced these physiologic changes, by reducing the enhancement of SGCs, ameliorating edema in the neurons, and normalizing the shape of the neurons. Therefore through relieving the neuronal stress state, ALA proved to have a neuroprotective effect.

Our findings using P53 immunohistochemistry also strengthened the evidence of that. P53 protein is a crucial player in the cellular response to neuronal stress [30]. P53 staining is employed as a biomarker of P53-induced apoptosis [31,32]. The P53 gene has been shown to play a vital modulatory role in neuropathic pain induced by DRG trauma [33]. Similarly, we found that P53 expression was significantly upregulated in the CCI rat model, paralleling the morphologic changes. This indicates that CCI promotes cellular apoptosis. As with improvement in morphologic changes, ALA treatment significantly reduced the number of P53+ cells. This is further evidence that ALA can ameliorate CCI-induced damage mediated by P53-induced apoptosis.

Although our study provided several different types of evidence of the efficacy of ALA in this model of neuropathic pain, the mechanisms by which it functions need further investigation. Reducing P53-induced apoptosis is one potential mechanism. It has been shown that neuronal apoptosis serves as a mechanism to maintain neuropathic pain, while suppression of cellular apoptosis ameliorates hyperalgesia and mechanical abnormal pain [34], which completely agrees with our findings in the present study. The mechanisms underlying cellular apoptosis may be closely associated with OS [34,35,36]. Battisti et al. used ALA to treat chronic low back pain, finding that ALA and superoxide dismutase (SOD) exhibited a synergistic effect to reduce neuropathic pain. This suggests that ALA might have a neuroprotective effect at least in part by reducing the OS that would otherwise cause neuropathic pain [36]. Using ALA to treat mechanical PNI has been shown to activate SOD and catalase [37]. All the evidence strongly suggests that OS-related mechanisms may be critically important in the neuroprotective effects of ALA on the neuropathic pain associated with PNI. Future study is required to further investigate these mechanisms.

There are several limitations in this study. First, we did not perform immunohistochemical staining for SGCs. Although using the methods introduced by Manzhulo et al. [20] could identify the SGCs, we believe in using the immunohistochemical staining for which SGCs labeled with the antibody will achieve better identification. Second, we used 50 mg/kg as the dose of ALA. Although no animal died in this study, previous study in diabetic rats reported that diabetic rats died in this dose [18]. Hence, 50 mg/kg is an extremely large dose for rats, which might be not the most appropriate for CCI. Unfortunately, we did not perform a preliminary experiment to determine the most optimal dose of ALA for CCI. All these limitations should be addressed in our future investigation.

## 5 Conclusions

Taken together, our study indicates neuroprotective effects of ALA on chronic neuropathic pain in a CCI rat model. Our results imply that ALA can be considered as a potential therapy for the neuropathic pain associated with PNI. Further verification with clinical testing, as well as exploring the therapeutic mechanisms, is required.
